# Lentiviral and Moloney Retroviral Expression of Green Fluorescent Protein in Somatotrophs *In Vivo*

**DOI:** 10.1371/journal.pone.0054437

**Published:** 2013-01-16

**Authors:** Masayoshi Okada, Hiroko Matsuda, Yasuhiko Okimura

**Affiliations:** 1 Department of Physiology, Kansai Medical University, Moriguchi City, Osaka, Japan; 2 Kobe Women’s University, Kobe, Hyōgo, Japan; Western University of Health Sciences, United States of America

## Abstract

Previous studies have shown that the locus control region (LCR) and the promoter of the growth hormone (GH) gene can control the expression of GH. Therefore, lenti- and retro-viral vectors with these elements might be useful to monitor the activation of the GH gene and the development of newborn somatotrophs. To test this, we first constructed a lentiviral vector, which expresses green fluorescent protein (GFP) under the control of these elements, and injected them into rat pituitaries in situ and in vivo. The lentiviral vector expressed GFP specifically in the anterior lobe, and nearly all GFP-positive cells were anti-GH immunoreactive. The GFP expression was upregulated by the administration of growth hormone releasing hormone and an IGF-1 receptor blocker. Furthermore, the social isolation stress, which was shown to decrease the GH secretion, decreased the GFP expression. Second, we injected the retroviral vector into neonatal rat pituitaries in vivo. At 30 days postinjection (DPI), almost all GFP-positive cells were anti-GH positive and anti-prolactin negative as the lentiviral expression. However, GFP was transiently expressed by developing lactotrophs at 8 and 16 DPI, suggesting that our vector lacks an element(s) which suppresses the expression. Meanwhile, the retrovirally labeled cells tended to cluster with the cells of same type. An analysis of cell numbers in each cluster revealed some features of cell proliferation. These viral vectors are shown to be useful tools to monitor the activation of the GH gene and the development of somatotrophs.

## Introduction

Somatotrophs secrete the growth hormone (GH), which plays pivotal roles in regulating physical growth and the metabolism of fatty acid and glucose. The anterior lobe of the pituitary contains five types of hormone-secreting cells, i.e., somatotrophs, lactotrophs, thyrotrophs, gonadotrophs, and corticotrophs. These cell types arise from a common primordium. In particular, somatotrophs and lactotrophs arise through a common cell lineage determined by transcription factors Prop-1 and Pit-1 whereas corticotrophs and gonadotrophs arise from a Pit-1-negative lineage [1]. Although the full set of those hormone-secreting cells are already differentiated at birth, their proliferation and differentiation continue even in the postnatal period [2], [3]. However, details of their postnatal development are largely unknown.

The human GH gene cluster contains one GH gene, which is specifically expressed in the pituitary, and four paralogues, which are expressed in the placenta. The transcription of GH mRNA is controlled by the locus control region (LCR) and the promoter of the gene [4]. The GH LCR regulates the tissue-specific expression of GH. Reportedly, the −14.5 to −32 kb region flanking the hGH promoter has five DNase I-hypersensitive sites (HSs). Of these five HSs, HSI was shown to be essential for pituitary-specific GH gene expression [5], [6]. Indeed, studies with transgenic mice have shown that a 404-bp region of HSI, linked to the GH promoter, recapitulates the pituitary-specific expression of the GH gene. The 404-bp region has three Pit-1 binding elements, which seem to play an essential role in the specificity [6], [7]. The expression of GH is regulated hormonally, e.g., the mRNA for GH is increased by growth hormone releasing hormone (GHRH) and decreased by IGF-1 as a negative feedback. The GH promoter was shown to be activated by the cAMP and cAMP response element-binding protein (CREB) pathway, which is stimulated by GHRH [8], and to be suppressed by the tyrosine kinase pathway, which is stimulated by IGF-1 [9]. Therefore, it is assumed that the GH LCR and GH promoter are the necessary and sufficient elements for the transcriptional control of GH gene. In addition, GH secretion is suppressed by psychosocial stress in humans [10], and this suppression can be reproduced in infant rats by maternal deprivation [11]. But, it is unknown whether these LCR and promoter can respond to psychosocial stress.

Lentiviral and Moloney retroviral vectors are derived from the retroviruses HIV and Moloney murine leukemia virus, respectively. Whereas the lentiviral vector can infect postmitotic cells, the Moloney retroviral vector can infect only mitotic cells. These two vectors, however, express the exogenous gene in a similar way. Following internalization of the viral particle, the reverse-transcribed DNA fragments are integrated into the genome of host cells. Since endogenous promoter activity has been eliminated from the long terminal repeat in the DNA of the latest version of the viral vectors, the expression of the exogenous gene is controlled by the promoter inserted within the viral vectors [12], [13].

In this report, to test whether the viral vectors can be used to monitor the activation of the GH gene and the development of pituitary cells, we prepared lentiviral and Moloney retroviral vectors that express green fluorescence protein (GFP) under the control of the GH LCR and promoter. The lentiviral vector successfully expressed GFP in a somatotroph-specific way, and the expression responded to the administration of GHRH and an IGF-1 blocker. The GFP expression was suppressed by psychosocial stress, isolation of neonatal rats. The Moloney retroviral vector expressed GFP in newborn somatotrophs. But unexpectedly, GFP was also expressed in developing lactotrophs, although only transiently. These data shows the usefulness of these vectors to monitor the activation of the GH gene and a difference in the control the GH gene between mature and newborn cells. We also discuss about the proliferation of retrovirally infected newborn somatotrophs.

## Materials and Methods

### Viral Vector Preparation

**Figure 1:**
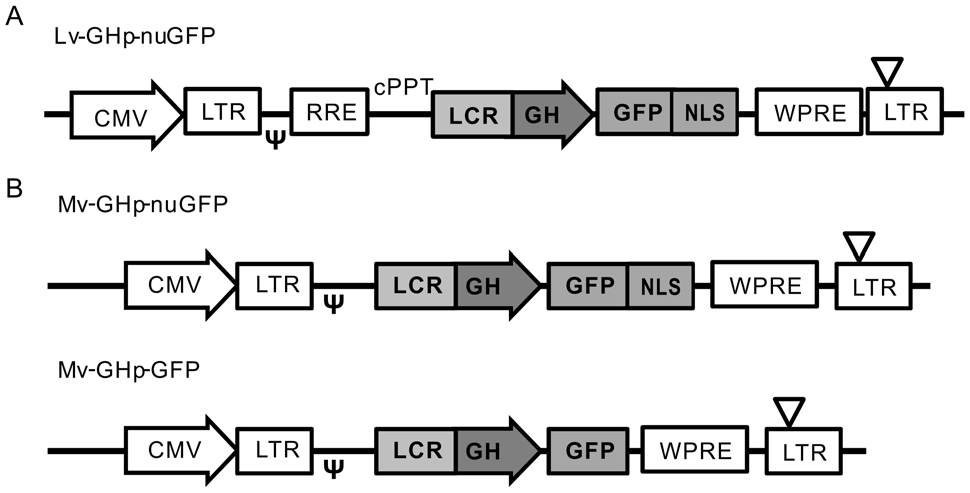
Schematic illustration of lenti- and retro-viral vectors. A. Structure of the Lv-GHp-nuGFP. B. Structure of Mv-GHp-nuGFP and Mv-GHp-GFP. The upstream CMV promoter was used to transcribe the viral genomic RNAs. CMV, cytomegalovirus promoter; LTR, long terminal repeat; *ψ*, RNA packaging signal; RRE, Rev responsive element; cPPT, central polypurine tract: LCR, locus control region; GHp, promoter of GH gene; GFP, green fluoresent protein gene. NLS, nuclear localization signal; WRRE, woodchuck hepatitis virus posttranscriptional regulatory element; ∇, deletion in the U3 region of the 3′ long terminal repeat.

The 404-bp fragment of the LCR was prepared by PCR with a set of primers, 5′-AACTGCAGCTTTGGGGAGACACTAGCCCCAAAGTTA-3′ and 5′-TTCTGCAGATCTTGGCCTAGGCCTCGGACCTGA-3′, and human genomic DNA. We added a Pst-1 site (underlined) to facilitate the insertion upstream of the GH promoter region (0.5 kb) of the GH-Luc plasmid [14], and confirmed the sequence. The lentiviral and Moloney retroviral vector plasmids were generously donated by Dr. Hiroyuki Miyoshi (Riken Tsukuba Institute, Ibaraki, Japan http://dna.brc.riken.jp/index.html) and Dr. Carlos Lois (University of Massachusetts, Worcester, MA) [15], respectively. Promoters in the vectors were replaced with the 404-bp LCR and the promoter of GH described above. The cDNA of hrGFP (Agilent Technologies Inc., Santa Clara, CA) or that fused with nuclear localization signal (NLS) of SV40 large T-antigen (nuGFP) was cloned downstream of the promoter (Fig. 1). The nucleotide sequence for the NLS was GAT CCA AAA AAG AAG AGA AAG GTA GAT CCA AAA AAG AAG AGA AAG GTA GAT CCA AAA AAG AAG AGA AAG GTA [16]. The construction of these promoter and cDNA was confirmed with restriction enzyme mapping and partial sequencing. The viral vectors were pseudotyped by VSV-g protein [17]. We prepared the viral vectors by calcium-phosphate transfection of the plasmids to 293T cells. We harvested the medium 48 h after transfection, and removed cell debris by centrifugation (1000× *g*, 3 min at 4°C) and filtration with a 0.45-µm filter. The viral particles were concentrated with centrifugation (58,000× *g*, 2 h at 4°C) and suspended in a small volume of PBS [13], [18]. The typical titer was 1×10^5^ infectious particles per microliter.

### Animals and Stress Loading

Sprague-Dawley rats of both sex were purchased from Clea-Japan (Tokyo, Japan). Rats were maintained on a 12∶12 light-dark cycle (8 AM–8 PM). Rats were anesthetized with inhalation of 5% isoflurane before fixation. The animals were fixed and administrated with GHRH and an IGF-1 receptor blocker, H-1356, at 10 AM. Animal and gene recombination experiments were approved by the committees of Kansai Medical University. All efforts were made to minimize the number of animals used and their suffering.

To test the effect of psychosocial stress on the expression of GFP, rats (P9-P16) were put into plastic cups separately, which were immersed in a water bath incubator circulating warmed water (34°C) from 9AM to 12PM. We weighed rats before the isolation.

### In Situ Expression in Organotypic Culture

An organotypic culture of the pituitary from postnatal day 7 (P7) rats was prepared according to methods from previous reports [19], [20]. Rats ware anesthetized with 5% isoflurane. Pituitaries were sagittally sliced with a surgical scalpel (300–400 µm in thickness), placed on culture insert membranes (Millicell®CM-ORG, Merck, Whitehouse Station, NJ), and cultivated in a 6-well plate supplemented with 1 ml of medium at 34°C [21], [22]. The culture medium contained 50% MEM, 25% HBSS, and 25% horse serum supplemented with glucose (5.5 g/l) and penicillin/streptomycin. Media were changed twice a week. Alternatively, whole pituitaries were placed in a Millicell membrane with the neural lobe up. Two days after tissue preparation, we injected the viral solution (0.1–0.2 µl/5-7 sites/slice) using a Femtojet micro injector (Eppendorf, Hamburg, Germany) as previously described [23]. Images were taken with an inverted microscope (IX71; Olympus, Tokyo, Japan).

### In Vivo Expression

For in vivo labeling, the viral vectors were injected into the P8 rat’s pituitary according to methods of previous studies [24], with some modifications. Pups, which were anesthetized on ice, were placed on a stereotaxic apparatus SR-5M (Narishige, Tokyo, Japan). To minimize the possibility of injection failure, we injected the viral solution to five sites (5 µl×5 sites) using a 30-G needle and a microsyringe (Ito Corporation, Shizuoka, Japan) from the dorsal side through the skull. The stereotaxic coordinates relative to Bregma was lateral 0.0 mm, and anterior-posterior 0.5, 0.0, −0.5, −1.0, and −1.5 mm. The needle was lowered until reaching the sphenoidal bone (ventral, about 10.0 mm). The incisor bar was located 3 mm above the ear bar. Viral solution was infused by pressing the cylinder with a finger for 30–40 s, and let stand for a few minutes before the removal of the needle. We did not observe any lethal effect of injection. After the suture of the scalp incision with super-glue, rats were allowed to recover on a heating pad (37°C) and returned to home cage as soon as the body temperature normalized.

### Immunostaining

The pituitaries of rats were fixed by transcardiac perfusion of 4% paraformaldehyde in PBS and sagittally sliced (75 µm in thickness) with a microslicer (PRO7, Dosaka, Kyoto, Japan). The slices were reacted with anti-GH (x2,000, Merck, Whitehouse Station, NJ), anti-prolactin (x1,000, Serotec, Oxford, UK), and anti-ACTH (x400, Acris, Herford, Germany) antibodies dissolved in PBS containing 0.3% BSA and 0.3% Triton-X-100, at 4°C for 48 h. The immunoreaction was visualized with a secondary antibody (x750, Alexa Flour 568; Invitrogen, Carlsbad, CA) at room temperature for 2 h. Images were taken with a confocal microscope (FV300; Olympus, Tokyo, Japan) and analyzed with ImageJ software. We used dichronic filters for EGFP and Texas red. The confocal images of lentiviral vectors are single plane images. Several Z plane images were stacked for the retroviral vector infected pituitaries, because of the small number of the positive cells.

For double immunostaining, we used antibodies against prolactin raised in guinea pig (5000×, The National Hormone and Peptide Program, Torrance, CA) and Cy5-labeled secondary antibody (500x, The Jackson Laboratory, Bar Harbor, ME), in addition to anti-GH antibody described above. Images were taken with a confocal microscope (LSM510; Carl Zeiss, Oberkochen, Germany).

### Statistical Analysis

Data are shown as mean ± SEM. We used Student’s *t*-test for statistical analysis, unless indicated otherwise in the figure legend. A p value of <0.05 was considered significant.

## Results

### Lentiviral Expression of GFP in Somatotrophs

**Figure 2:**
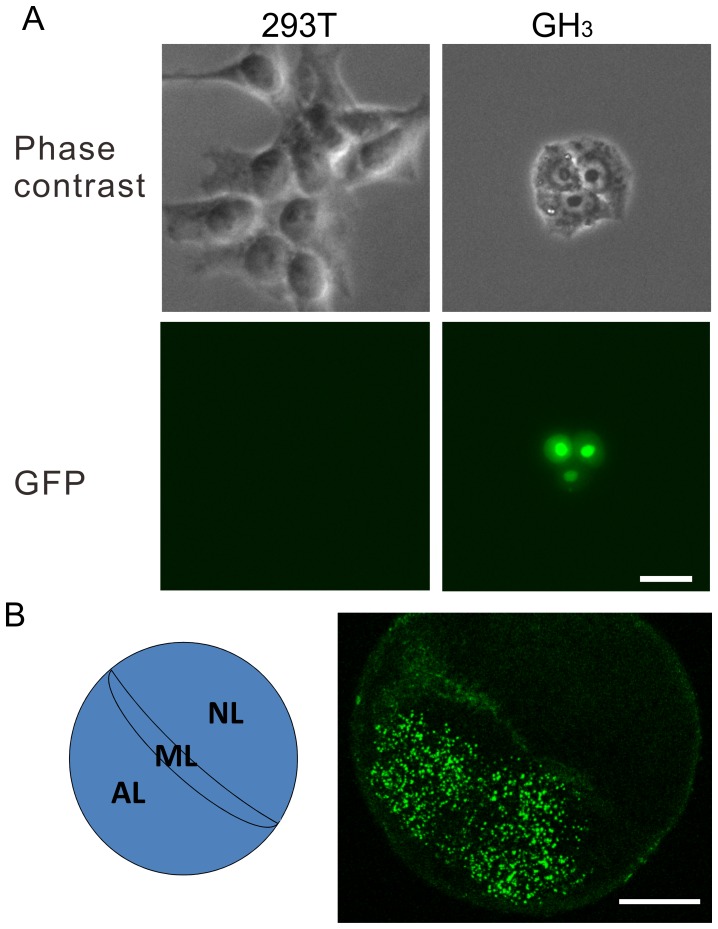
Specificity of lentiviral expression of GFP in vitro and in situ. (A) Lentiviral expression observed in GH_3_ cells but not in 293T cells. Lv-GHp-nuGFP was added to the media of 293T and GH_3_ cells. Images were taken 48 h later. nuGFP was expressed only in GH_3_ cells, and the nuclear localization of the fluorescence indicates the functioning of the NLS attached to GFP cDNA. Bar, 20 µm. (B) Lentiviral expression in the anterior lobe in an organotypic culture. Two days after cultivation of a sagittal slice of the rat pituitary, Lv-GHp-nuGFP was injected into both the anterior lobe (AL) and the neural lobe (NL). ML, medial lobe. The expression of GFP was observed only in the anterior lobe 7 DPI. Bar = 0.2 mm.

We prepared a lentiviral vector that contains the GH LCR and the promoter upstream of GFP gene attached with nuclear localization signal (referred to as Lv-GHp-nuGFP)(Fig. 1A). To test the specificity of the expression, we infected human embryonic kidney 293T cells and GH_3_ cells with Lv-GHp-nuGFP. Expectedly, GFP was expressed only by GH_3_ cells, which endogenously express GH (Fig. 2A).

To test the specificity in situ, we prepared an organotypic culture of a sagittal slice of the pituitary and injected Lv-GHp-nuGFP into both the anterior and the neural lobes. Since the slice was cultivated on a membrane, we could identify the injection site in the anterior and the neural lobes. Seven days postinjection (DPI), GFP expression was observed only in the anterior lobe (Fig. 2B).

**Figure 3:**
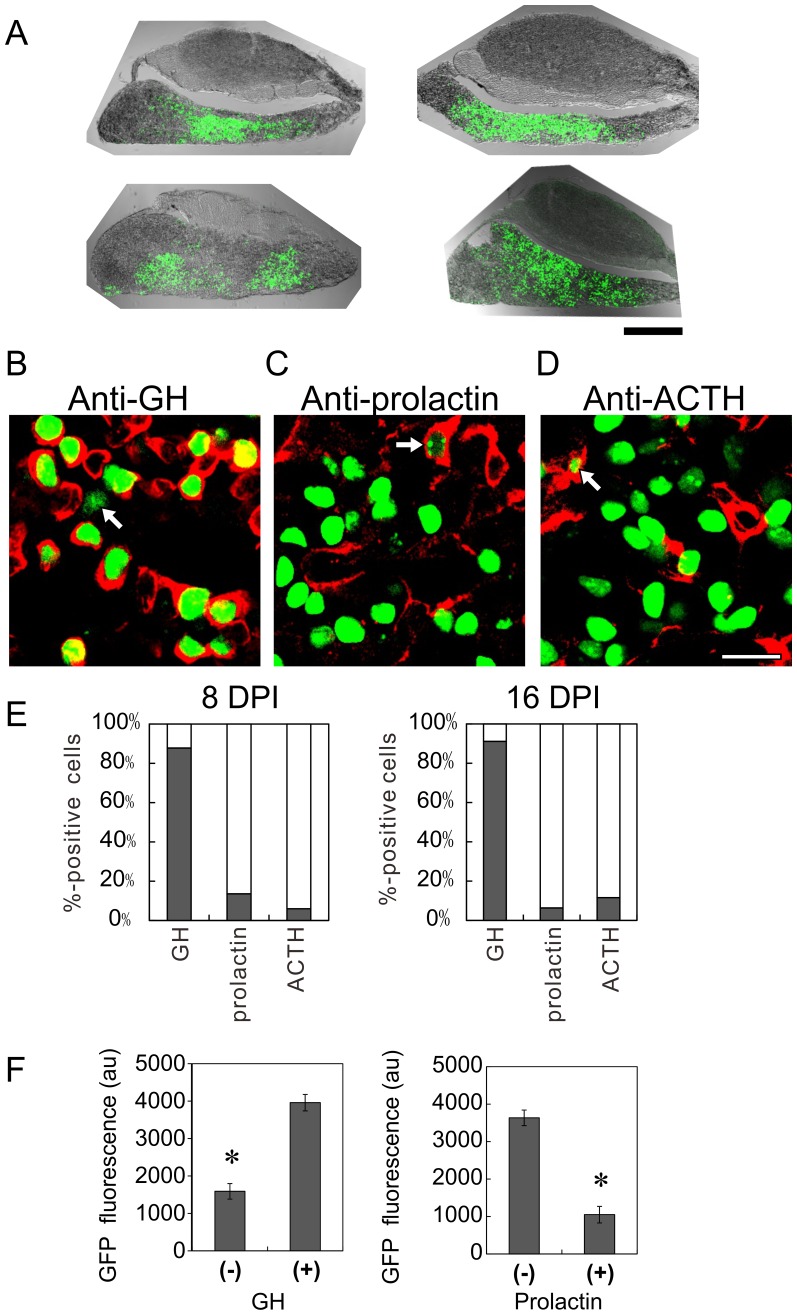
In vivo lentiviral expression of GFP. (A) Typical confocal images of the pituitaries injected with Lv-GHp-nuGFP from four rats. We fixed the pituitaries at 8 DPI and sliced them sagittally. Bar, 0.5 mm. (B–D) Somatotroph-specific expression of GFP in Lv-GHp-nuGFP-injected rat pituitary. The pituitary slices, prepared at 8 DPI, were immunostained with anti-GH (B), anti-prolactin (C), and anti-ACTH (D) antibodies. The immunoreactions were visualized with Alexa-569 (red fluorescence). Note the green fluorescence of GFP in the nuclei and the surrounding red anti-GH immunoreactivity. Most GFP-positive cells were GH positive at 8 DPI. Only a few GFP-expressing cells were GH negative (arrow in B). In contrast, most GFP-positive cells were prolactin and ACTH negative, but only a few were prolactin and ACTH positive (arrows in C and D). Bar, 20 µm. (E) Percentages of GH-, prolactin-, and ACTH-positive cells at 8 and 16 DPI. We analyzed 100 GFP-expressing cells from 2 rats. (F) Lower GFP expression in the GH-negative and prolactin-positive cells. The expression of GFP was analyzed with ImageJ software and was indicated as the arbitrary unit (au) of the software. The expressions of GFP were lower in GH-negative and prolactin-positive cells (n = 10, p<0.00001).

Previously, viral vectors were successfully injected into the pituitary via transauricular [25] or dorsal [24] approach. We then injected the lentiviral vector into the pituitary of P8 rats by using a stereotaxic apparatus from the dorsal side. The pituitaries were fixed at 8 DPI and sagittally sliced. Robust GFP expression was observed in the anterior lobe (Fig. 3A). To secure a successful injection, we injected the viral solution at five sites along the midline, and thereby one or two densely GFP-positive areas were observed.

To test the cell type specificity of GFP expression, the slices were immunostained with anti-GH (Fig. 3B), anti-prolactin (Fig. 3C), and anti-ACTH (Fig. 3D) antibodies. Expectedly, 87.8% of GFP-expressing cells were GH positive at 8 DPI, and similar results were obtained at 16 DPI (91.1%; Fig. 3E). A few GFP-expressing cells were anti-prolactin positive (13.6% at 8 DPI, 6.3% at 16 DPI) and anti-ACTH positive (5.9% and 11.6%).

As shown in Fig. 3B and 2C, GFP expression in GH-negative cells (arrows) was lower than those in the other cells. For statistical analysis, we measured the nuclear GFP fluorescence with ImageJ software, and found a significant difference in GFP expression (Fig. 3F). Similarly, the expression level was significantly lower in prolactin-positive cells (Fig. 3C and F). These results suggest that our lentiviral vector substantially retains cell type specificity in matured cells.

### Response of Lentiviral Expression of GFP to Hormonal and Environmental Stimulation

**Figure 4:**
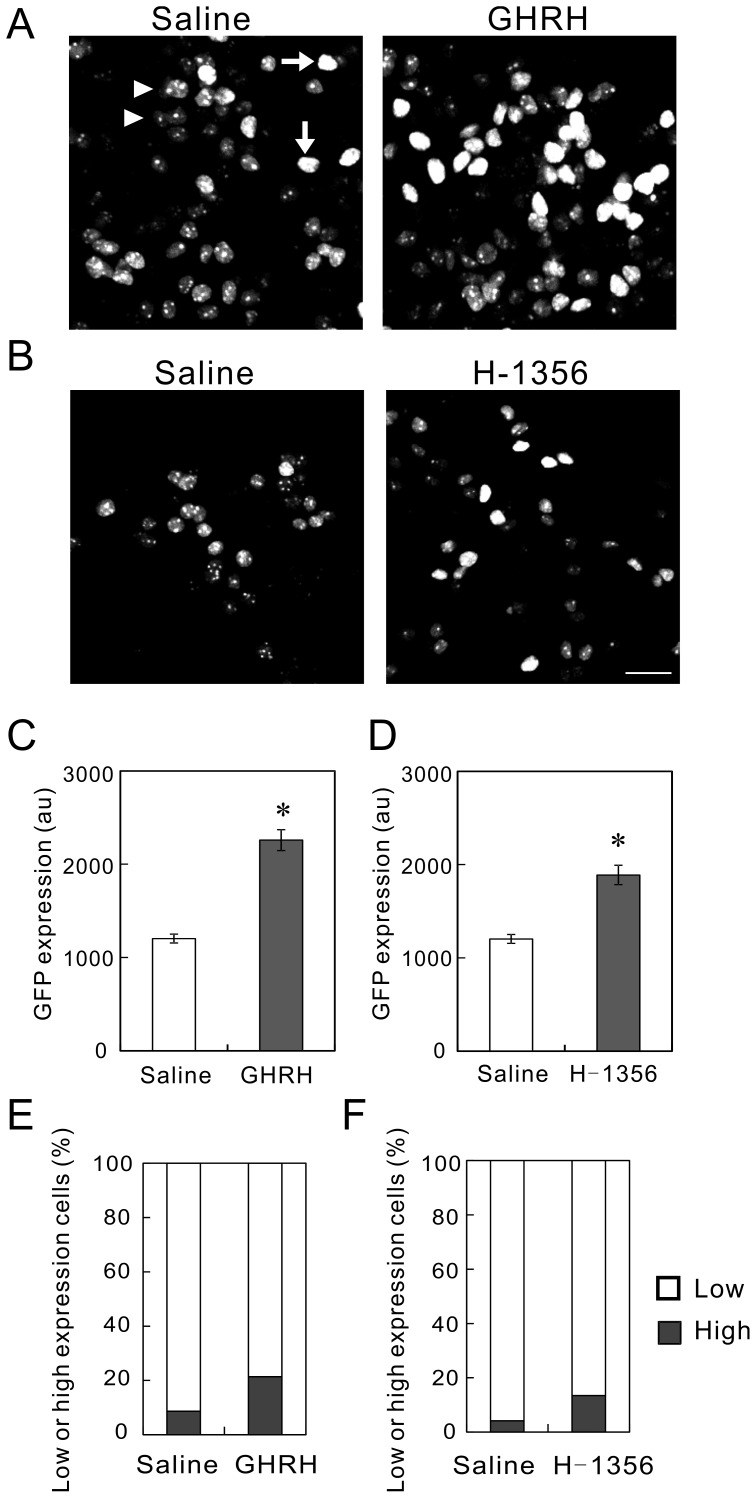
Increase in GFP expression in response to GHRH and an IGF-1 receptor blocker. (A) GHRH-induced increase in GFP expression. GHRH was administrated for 3 days from 9 DPI. The pituitaries were fixed 24 h after the last injection and then sagittally sliced. We took images with a confocal microscope. The GFP-expressing cells were classified as low-expression cells (arrowheads) and high-expression cells (arrows). (B) IGF-1 receptor blocker-induced increase in GFP expression. H-1356, a blocker of the IGF-1 receptor, was administrated for 2 days. Bar, 50 µm. (C) Increase in GFP expression induced by GHRH. GFP expression in the nuclei was analyzed with ImageJ software, and was indicated as the arbitrary units (au). The expression was significantly increased by the administration of GHRH (p<0.005, 30 cells/rat, n = 4). (D) Significant increase in GFP expression induced by H-1356 (p<0.05, 30 cells/rat, n = 4). (E and F) Increase in the percentage of GFP-high-expression cells induced by GHRH and H-1356. The statistical analysis showed a significant increase in GFP expression by GHRH (p<0.000005, χ^2^-test, totally 850 cells from four rats) and by H-1356 (p<0.000005, χ^2^-test, totally 600 cells from four rats).

Since the expression of GH is increased by GHRH, we tested the response of GFP expression to GHRH. We administrated GHRH (1 mg·kg^−1^day^−1^, i.p.; Peptide Institute, Osaka, Japan) for 3 days from 8 DPI, and fixed the pituitary 24 h after the last administration. Confocal images showed that GHRH administration increased the expression of nuGFP compared with saline administration (Fig. 4A). We measured the nuclear GFP fluorescence and found a significant increase due to GHRH (Fig. 4C). We then categorized the expression of nuGFP as “high expression,” in which the nucleus is evenly bright (arrows) or “low expression,” in which there are bright spots (arrowheads). The percentage of high-expression cells was significantly increased by GHRH (Fig. 4E).

We next examined the response to an IGF-1 receptor blocker, H-1356 [26], since rats were more sensitive to IGF-1 receptor blocker rather than IGF-1 itself (Unpublished data). H-1356 (Calbiochem, San Diego, CA) was administrated for 2 days (3 mg kg^−1^ day^−1^, i.p.), and pituitary slices were prepared as previously described. Since IGF-1 downregulates GH expression as a negative feedback, H-1356 should increase the expression. Expectedly, the fluorescence of nuGFP was increased by H-1356 (Fig. 4B and D); the fraction of high-expression cells was significantly higher than that in the saline control (Fig. 4F). These data suggest that our lentiviral vector retains the hormonal response to GHRH and IGF-1 in matured cells.

**Figure 5:**
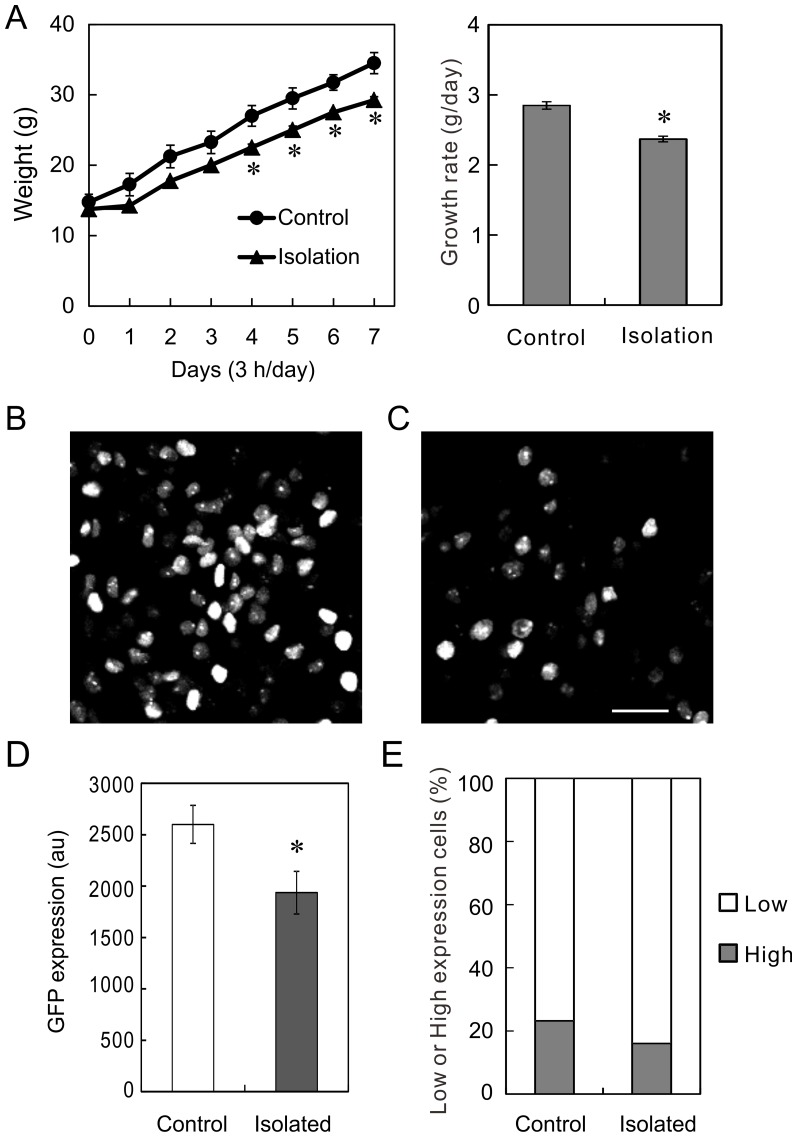
Psychosocial stress a decreases the GFP expression in vivo. (A) Slow growth of isolated rats. Neonatal rats were isolated from mother and siblings (3 h/day) for 7 days. Isolation significantly reduced the weight (p<0.05, n = 4) and the growth rate (p<0.0005, n = 4). (B and C) GFP expressions in the control (B) and isolated (C) rats pituitaries. Bar = 20 µm. (D and E) Isolation induced decrease in the GFP expression. (D) The GFP expressions in nuclei were analyzed with ImageJ software. The ordinate indicates the arbitrary unit of the software averaged from 12 to 30 cells. The isolation significantly decreased the GFP expression (p<0.05, n = 4). (E) The GFP positive cells were classified as low and high expression cells. The percentage of high expression cells significantly decreased in isolated rats (p<0.05, totally 600 cells, n = 4, χ^2^-test).

Reportedly, the growth of neonatal rat is dependent on the rearing environment, and impaired by psychosocial stress. To test whether our viral vector responds to the psychosocial stress, we isolated the Lv-GHp-nuGFP-injected rats in plastic cups (3 h/day for 7days). We weighed the rats every day and found the growth of isolated rats was slower than that of control rats (n = 5, Fig. 5A). Then we fixed the pituitaries and examined the expression of GFP (Fig. 5B and C). We compared the GFP expressions of 30 cells and found that isolation reduced the expression (Fig. 5D) and the percentage of high expression cells (Fig. 5E).

### Moloney Retroviral Expression of GFP in Developing Cells

**Figure 6:**
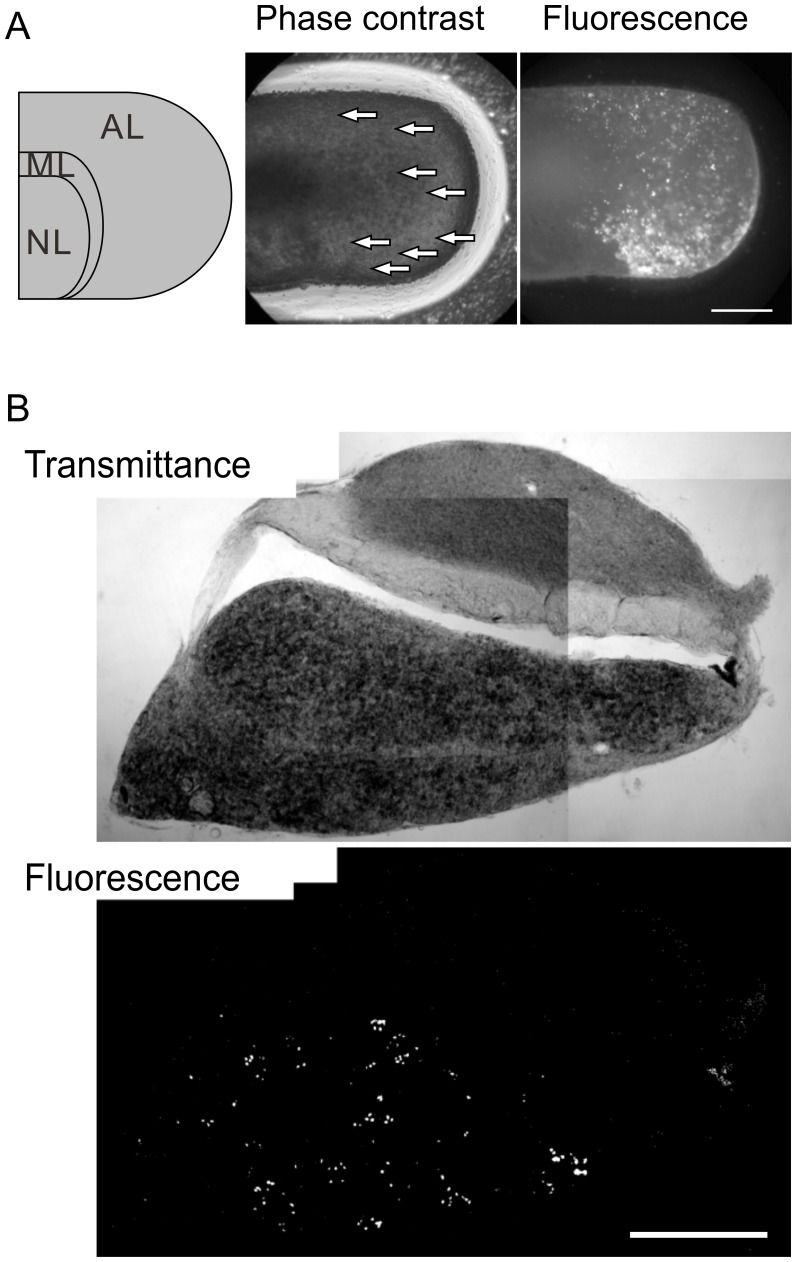
GFP expression in newborn somatotrophs in situ and in vivo. (A) GFP expression in organotypic culture. A whole pituitary was mounted on a milli-cell membrane and cultivated. The Schematic illustration and the phase contrast image shows that the culture retains the basic structure of the neural lobe (NL), the medial lobe (ML), and the anterior lobe (AL). Mv-GHp-GFP was injected into the anterior lobe. The arrows indicate the appropriate position of the injection sites according to our handwritten note. The fluorescence images of the same culture show GFP-expressing cells around the injection sites, suggesting that proliferation in the anterior lobe. Bar, 0.5 mm. (B) Retroviral GFP expression in vivo. The pituitary was fixed 16 days after the injection of Mv-GHp-nuGFP. GFP-expressing cells were sparsely located and clustered. Bar, 0.2 mm.

To test the retention of transcriptional control of our viral vector in newborn somatotrophs and to examine the development of newborn somatotrophs, we prepared Moloney retroviral vectors that express nuGFP (referred to as Mv-GHp-nuGFP) or GFP (referred to as Mv-GHp-GFP) under the control of the GH LCR and promoter (Fig. 1B). We injected the Mv-GHp-GFP into the anterior lobe of whole-mount organotypic cultures of the pituitary. At 8 DPI, GFP expression was observed around the injection sites (arrows) in the anterior lobe (Fig. 6A), suggesting cell proliferation in the anterior lobe.

We next injected Mv-GHp-nuGFP into the pituitaries of P8 rats, and the pituitaries were fixed at 16 DPI. GFP-expressing cells were observed sporadically in the anterior lobe (Fig. 6B). The number of GFP-positive cells was smaller than that in lentiviral labeling, despite similar titers of Mv-GHp-nuGFP and Lv-GHp-nuGFP, which were determined with GH_3_ cells.

**Figure 7:**
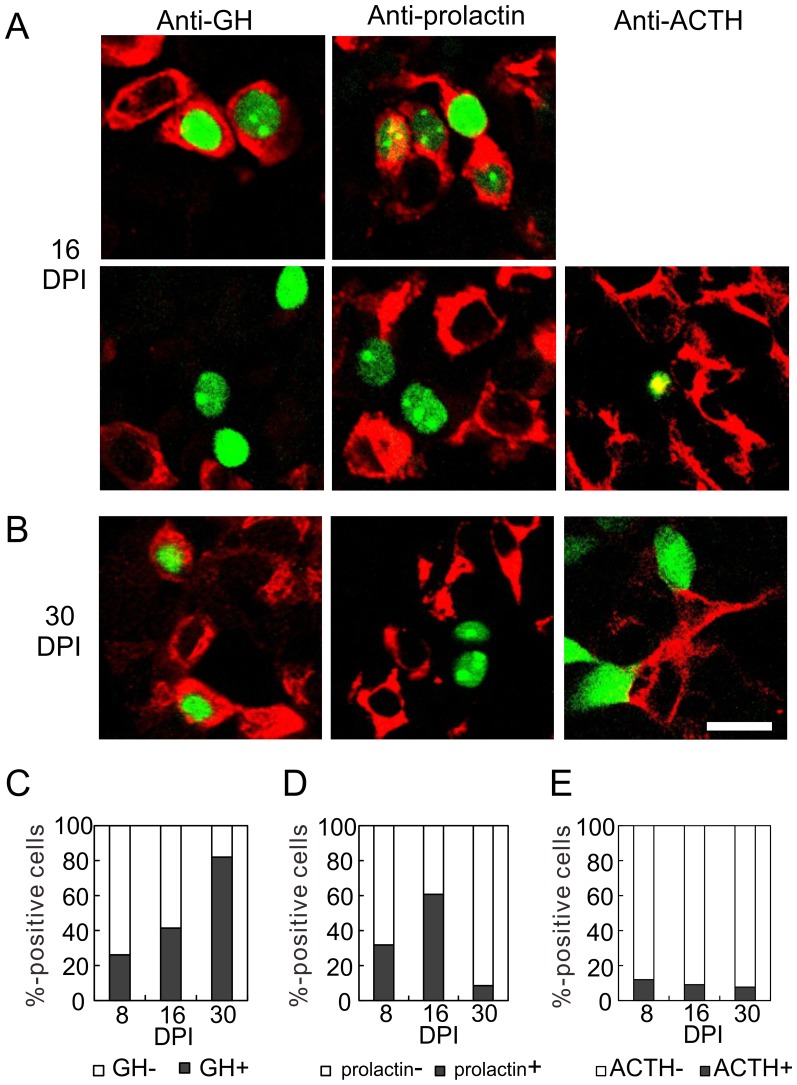
Cell type specificity of GFP-expressing newborn cells. We injected Mv-GHp-nuGFP or Mv-GHp-GFP into the pituitaries of P8 rats. Pituitary slices at 16 DPI (A) and 30 DPI (B) were immunostained with anti-GH, anti-prolactin, and anti-ACTH antibodies. (A) At 16 DPI, some nuGFP-positive cells were anti-GH positive (upper panel), but others were negative (lower panel). Similarly, there were anti-prolactin-positive (upper) and -negative cells (lower). The GFP-positive cells were hardly anti-ACTH positive (not shown) and mostly ACTH-negative (lower). (B) GFP expression in GH-positive cells at 30 DPI. Most GFP-expressing cells were GH-positive, and prolactin- and ACTH-negative. For the anti-ACTH staining, Mv-GHp-GFP was used instead of Mv-GHp-nuGFP. Bar, 20 µm. (C and D) Developmental changes in the percentages of GH-positive (C)(250 cells from four rats), prolactin-positive (D)(220 cells, n = 4), and ACTH-positive (E) (170 cells, n = 4) among the GFP-expressing cells. Note the constant increase in GH-positive cells and the transient increase in prolactin-positive cells.

To examine the temporal correlation between the expression of GFP and GH, we prepared pituitary slices at 8, 16, and 30 DPI, and immunostained them with an anti-GH antibody (Fig. 7A and B). Unexpectedly, only 26.1% and 41.4% of GFP-expressing cells were GH positive at 8 and 16 DPI, respectively (Fig. 7C). The percentage of GH-positive cells increased to 81.9% at 30 DPI, as cells matured, which is comparable to that in the lentiviral vector experiment.

To examine GFP expression in other cell types, we immunostained the pituitary slices with anti-ACTH and anti-prolactin antibodies. Only a few GFP-positive cells were anti-ACTH positive throughout the examination period (Fig. 7A and B). The number of anti-ACTH-positive cells was comparable to that in the lentiviral vector groups (compare Fig. 7E with Fig. 3E). Interestingly, 31.8% of GFP-expressing cells were prolactin positive at 8 DPI, which further increased to 60.7% at 16 DPI (Fig. 7D). Thereafter, the fraction of prolactin-positive cells decreased to 8.5% at 30 DPI, which is comparable to that in the lentiviral vector experiment.

**Figure 8:**
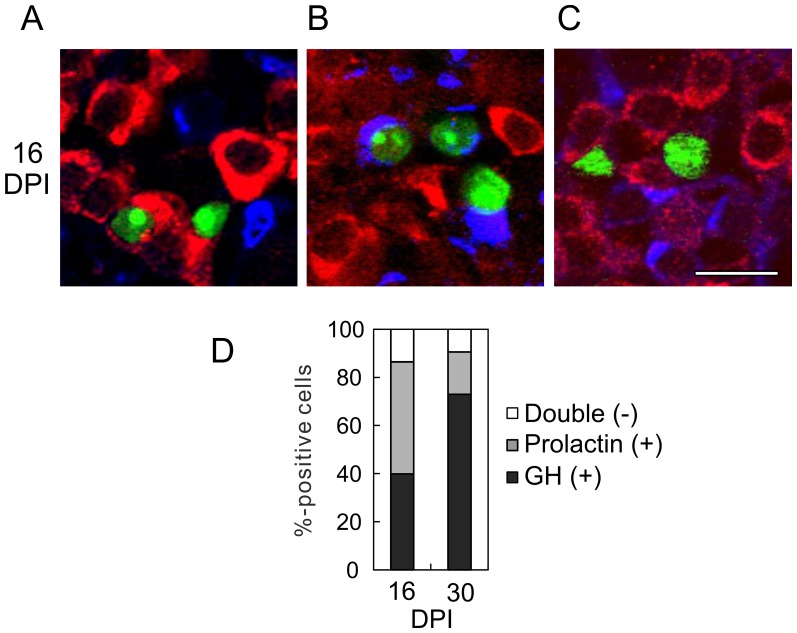
Anti-GH and anti-prolactin double immunostaining. Pituitary slices were prepared from Mv-GHp-nuGFP-injected rats at 16 DPI, and immunostained with anti-GH (red) and anti-prolactin (blue) antibodies. nuGFP-expressing cells were classified as anti-GH positive (A), prolactin positive (B), or double negative (C). None of the cells were GFP, GH, and prolactin triple positive. Bar, 20 µm. (D) Percentages of GH-positive, prolactin-positive, and double-negative cells at 16 and 30 DPI.

To test whether prolactin and GFP double-positive cells express GH, pituitary slices at 16 DPI were simultaneously stained with anti-prolactin and anti-GH antibodies. We observed GFP and GH double-positive cells (Fig. 8A) and GFP and prolactin double-positive cells (Fig. 8B), but no triple-positive cells. A few GFP-positive cells were GH and prolactin double negative (Fig. 8C and D). These results suggest that GFP was transiently expressed by the developing lactotrophs.

### Cluster Analysis

The GFP-expressing cells did not distribute evenly; they tended to cluster with several other cells (Fig. 6B). Assuming that a cluster was derived from a single Mv-GHp-nuGFP-infected cell, an analysis of these clusters should provide information about how these cells differentiate and proliferate. We defined cells within a cluster as follows: localization within 50 µm and a similar level of GFP expression.

**Figure 9:**
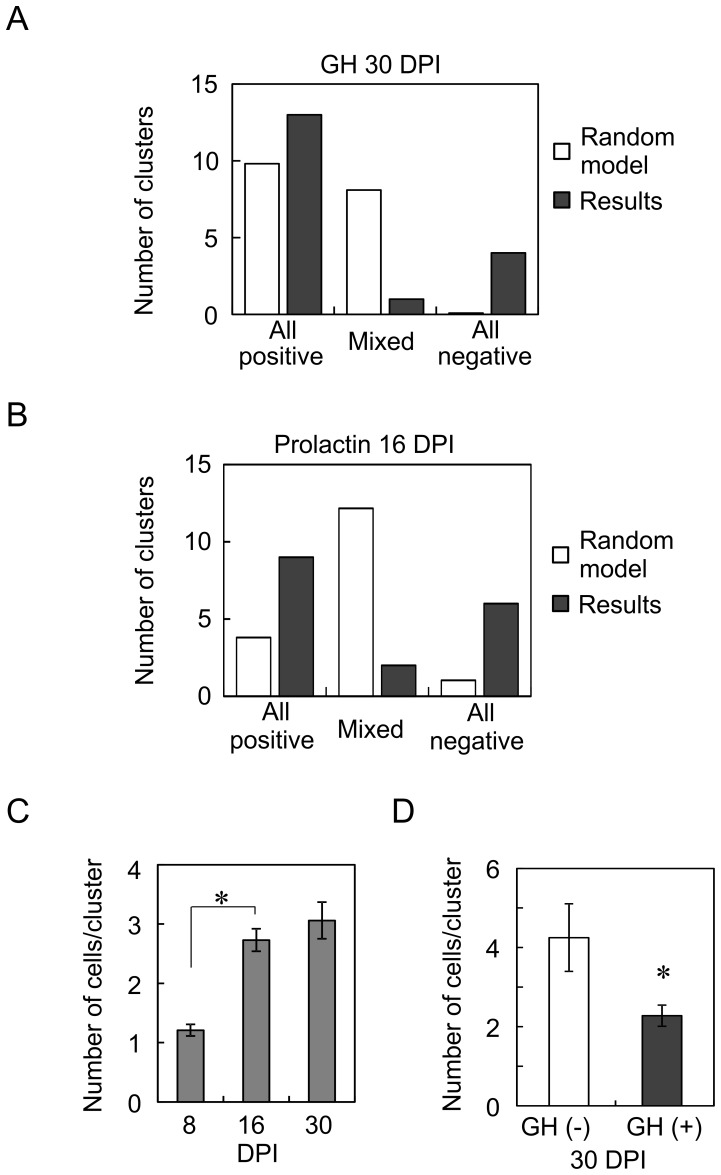
Cluster analysis of GFP-expressing cells. (A) Homogenous clustering of GH-positive and GH-negative cells. The open columns indicate the theoretical values according to the random clustering model of heterogeneous cells, and the closed columns indicate the results for GH at 30 DPI. Note that the numbers of all-GH-positive and all-GH-negative clusters were higher than those in the random model, and the number of mixed clusters was smaller than that in the random model. (B) Homogenous clustering of prolactin-positive and prolactin-negative cells at 16 DPI. Again, the numbers of all-prolactin-positive and all-prolactin-negative clusters were larger than those in the random model, and that of the mixed clusters was smaller than that in the random model. (C) Increase in cell number in each cluster. The cell number per cluster was significantly increased from 8 to 16 DPI, but the increase from 16 to 30 DPI was not significant (n = 18, 19, and 17, respectively; *, p<0.0000001, ANOVA). (D) A small cell number in the GH-positive clusters. The number of cells in each of the all-GH-positive clusters was significantly smaller than that of the all-negative clusters (n = 13 and 4, respectively; p<0.05).

We first counted the number of cells in each cluster at 8, 16, and 30 DPI. Most clusters consisted of single cells at 8 DPI. The numbers of cells in each cluster increased from 8 to 16 DPI, but, the increase in cell numbers from 16 to 30 DPI was not significant (Fig. 9C), suggesting a major proliferation period.

We next examined whether each cluster consists of homologous cells or not. We analyzed 23 clusters in total and classified them into all-GH-positive, mixed, and all-GH-negative clusters. If the heterogeneous GFP-expressing cells clustered at random, the postulated number of all-positive clusters would be postulated as 23 × (0.819)^3.06^ = 9.8, because 81.9% of GFP-expressing cells were GH positive and the mean cell number per cluster was 3.06 at 30 DPI. The experimental result was 13 (Fig. 9A). Similarly, the number of all-negative clusters was also higher than that in a random model. In contrast, the number of mixed clusters was smaller than that in the random model. We also analyzed anti-prolactin immunoreactive cells in each cluster at 16 DPI. The numbers of all-positive and all-negative clusters were again higher than the postulated values (Fig. 9B). The number of mixed clusters was lower than that in the random model. Clusters seemed to consist of homologous cells, supporting the assumption that each cluster was derived from a single infected cell. Furthermore, these results also suggest that the phenotype of GFP-positive cells, i.e., GH positive or GH negative, was determined before proliferation. We finally compared the number of cells in GH-positive clusters with that in GH-negative clusters at 30 DPI. The average cell number of GH-positive clusters was significantly lower than that of GH-negative clusters (Fig. 9D), suggesting slower proliferation in differentiated somatotrophs.

## Discussion

In this study, we prepared lentiviral and Moloney retroviral vectors that express GFP under the control of the GH LCR and promoter. The vectors specifically expressed GFP in the differentiated somatotrophs. The lentiviral GFP expression responded to hormonal stimuli and was suppressed by the psychosocial stress. These data showed that the GH LCR and promoter sufficiently controled the expression of GFP in mature somatotrophs. However, the GFP expression did not recapitulate the GH expression in immature cells: more than half of GFP-positive cells were GH negative at 8 and 16 DPI, and GFP was also expressed by lactotrophs at that period. The regulation seems to be different in immature cells, and other element(s) might be needed.

### Lentriviral Expression of GFP

The specificity of GFP expression of the lentiviral vector was evidenced by the following results: 1) GFP expression in GH_3_ cells but not in 293T cells; 2) GFP expression in the anterior lobe of organotypic pituitary culture and in vivo; and 3) colocalization of GFP with the anti-GH immunoreactivity in vivo. However, few GFP-expressing cells (approximately 10%) were prolactin and ACTH immunoreactive. This may be attributable to overlapping in the slices. In addition, the GFP gene might be activated by the promoter activity of the flanking region of the genomic integration site. The low expression of GFP in GH-negative and prolactin-positive cells supports this possibility.

Importantly, GFP expression responded to the administration of GHRH and H-1356, suggesting the retention of hormonal response in our GH-LCR and promoter construct [27]. Reportedly, GHRH stimulates GH gene expression through the activation of the GH promoter [28]. IGF-I inhibits GH gene expression through the inhibition of the GH promoter [9]. It is unclear whether the LCR plays a role in the hormonal regulation of GH gene expression. We think deletion of the LCR from the viral vector could address this question.

Reportedly, the psychosocial stress decreases the serum GH, but it is unknown whether the GH LCR and promoter are involved in the suppression. Our results showed the decrease in a GFP expression in isolated rats, suggesting that our viral vector can respond to the psychosocial stress. GH is secreted in a pulsatile way and the half-life of GH in blood is very short. Contrastingly, the half-life of GFP is 26 h [29]. Therefore, our GFP-based method seems to be suitable to detect chronic changes.

### Moloney Retroviral Expression

The Moloney retroviral vector successfully labeled the newborn cells in situ and in vivo. GFP-positive cells were observed in the anterior lobe of the organotypic cultures and in vivo, supporting the notion that postnatal proliferation occurs in the anterior lobe [30].

The Moloney retroviral vector expressed GFP in some developing lactotrophs at 8 and 16 DPI, although GFP was hardly expressed by the mature lactotrophs at 30 DPI, as in the lentiviral vector groups. Previous studies suggested that somatotrophs and lactotrophs are differentiated through a common intermediate stage, the mammosomatotroph, which coexpresses GH and prolactin [31]. However, others reported negative results for the presence of the mammosomatotroph [32]. In our results, none of the GFP- and prolactin-positive cells were GH positive at 16 DPI, suggesting that these cells were lactotrophs. Thus, it is likely that the transcriptional machinery of lactotrophs is closely related to that of somatotrophs, and thereby the GH LCR and promoter were transiently activated in the developing lactotrophs at least to some extent.

Then a question is arisen: why our vector transiently expressed GFP in developing lactotrophs? If it contains complete set of the elements, which are required for the regulation of expression, the expression of GFP should be completely the same as that of GH. But the results were different in the developing lactotrophs. There are two possibilities: First, the viral vector contains extra element which is activated in developing lactotrophs. This is unlikely, because the vector contains minimum elements, i.e. the LCR and promoter. Second, somatotrophs and developing lactotrophs may have common transcriptional machinery, and the expression of GH might be suppressed by an additional element. If our vector lacks the element, GFP should be expressed in developing lactotrophs. Thus it is likely that our viral vector does not contain the element(s), which probably regulates expression negatively in developing lactotrophs. Possibly, lactotrophs lose the common machinery during development, and thereby do not express GH as they mature. Although some negative elements have been reported within the GH promoter region [9], [33], our promoter construct contains those elements. Thus, additional element seems to be needed to suppress the expression in the developing lactotrophs. Further studies are needed to elucidate this regulatory element.

### Cluster Analysis

Moloney retroviral labeling showed the clustering of several GFP-positive cells. We assumed that these cells were derived from a single infected cell. These clusters can be classified into all-positive, mixed, and all-negative clusters. If the cell phenotype is determined before proliferation, the clusters will consist of homologous cells. Expectedly, the numbers of all-positive and all-negative clusters were higher than the postulated numbers in a random clustering model. Phenotype seems to be determined before proliferation.

The number of cells per cluster increased from 8 to 16 DPI, but did not from 16 to 30 DPI, suggesting a major proliferation period. Meanwhile, as shown in Fig. 8, a certain number of cells were GH negative even at 30 DPI. The cell number of the all-GH-negative cluster was significantly higher than that of the GH-positive cluster. Cell proliferation seems to be slower in the differentiated GH-positive cells. Probably, the GH-negative cells play the role of stem cells for the postnatal development of somatotrophs.

Our present data indicate that the Moloney retroviral vector is useful for investigating cell proliferation and differentiation in relation to the promoter, and has some advantages over the BrdU method: 1) no need for immunostaining, 2) the expression of GFP precedes the anti-GH immunoreactivity, and thereby makes an earlier analysis possible, 3) only a limited number of cells are labeled, which enables the analysis of clonally related cells, 4) whereas BrdU immunoreactivity decreases by half with each cell division, the GFP expression does not, and 5) this method is applicable to living cells and time-lapse imaging.
